# High-Grade Pure Esophageal Neuroendocrine Carcinoma Arising From Barrett’s Mucosa: A Rare Phenomenon

**DOI:** 10.7759/cureus.40644

**Published:** 2023-06-19

**Authors:** Parth Patel, Eli A Zaher, Hasan Sqour

**Affiliations:** 1 Internal Medicine, Ascension Saint Joseph Hospital, Chicago, USA

**Keywords:** endoscopic ultrasonography fine needle aspiration, endoscopic ultrasonography, esophageal neuroendocrine carcinoma (e-nec), barrett's esophagus (be), neuroendocrine carcinoma(nec)

## Abstract

Esophageal neuroendocrine carcinoma (E-NEC) is a very rare neuroendocrine tumor. There are only a few case reports where pure esophageal NEC is found to be arising from Barett's mucosa. Here we present a case of high-grade pure E-NEC arising from Barrett's esophagus, which was metastasized to the liver at the time of diagnosis.

## Introduction

Esophageal neuroendocrine carcinoma (E-NEC) is uncommon and has a poor prognosis. It predominantly affects men aged 60-70 and is strongly associated with tobacco and alcohol use [1]. Barrett's esophagus has been so far rarely linked with E-NEC. The chronic inflammation associated with Barrett's esophagus could potentially lead to forms of esophageal cancer other than the well-described adenocarcinoma [2]. Here we present a case of E-NEC in a 47-year-old male without a significant smoking or drinking history who had an 18-year history of Barrett's esophagus.

## Case presentation

**Figure 1:**
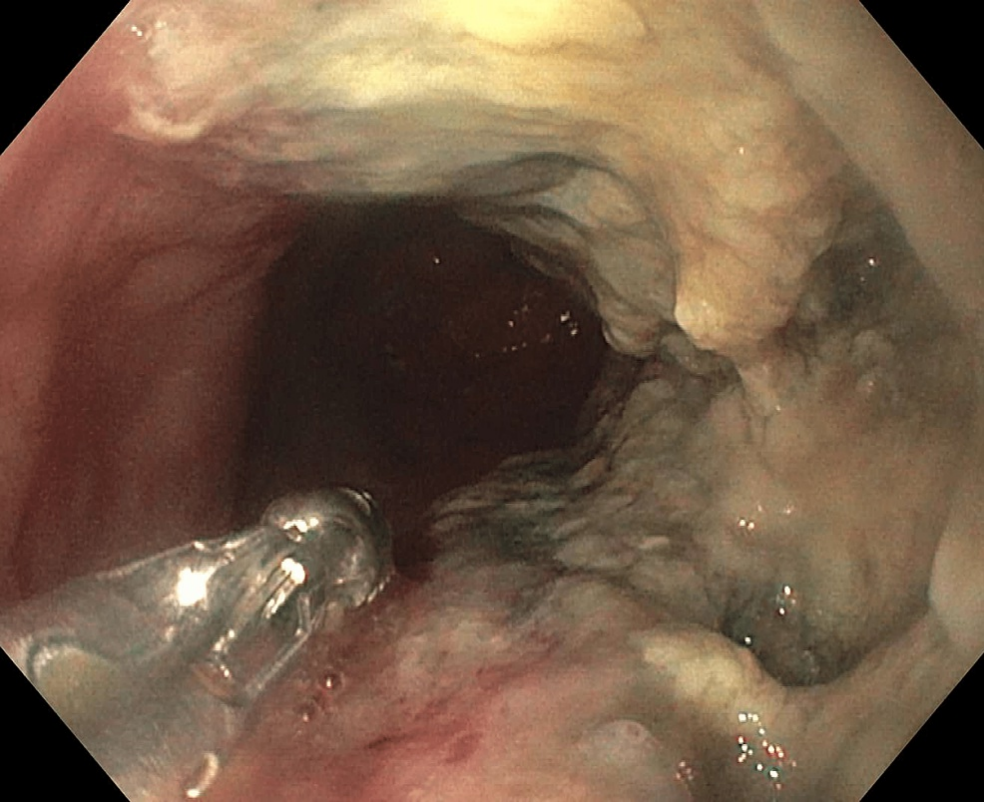
Esophagogastroduodenoscopy Mass in the lower esophagus

A 47-year-old male with an 18-year history of Barrett's esophagus presented with one week of worsening fevers, chills, dyspnea, bloody sputum, and odynophagia. CT angiography showed multifocal pulmonary emboli and circumferential thickening of the distal esophagus. Hence an upper endoscopy was done. It revealed a 5 cm fungating mass with ulcerations in the lower third of the esophagus and mucosal changes secondary to established long-segment Barrett's disease (Figure 1).

**Figure 2:**
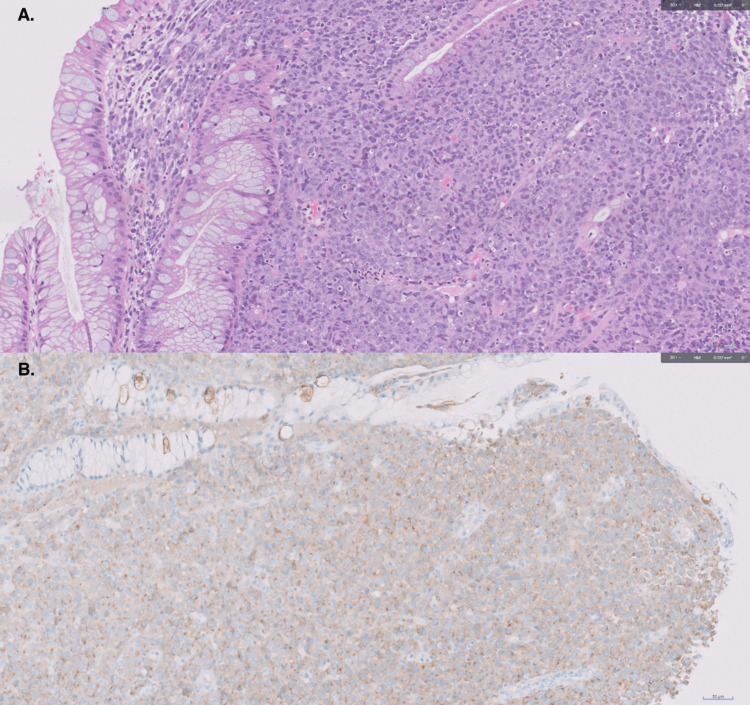
A: H&E stain. B: Synaptophysin stain. A: 20x view of esophageal mucosa with specialized goblet cell type intestinal metaplasia (Barrett's esophagus) and diffuse growth of neuroendocrine carcinoma B: 20x view showing lesional cells diffusely positive for neuroendocrine marker Synaptophysin.

**Figure 3:**
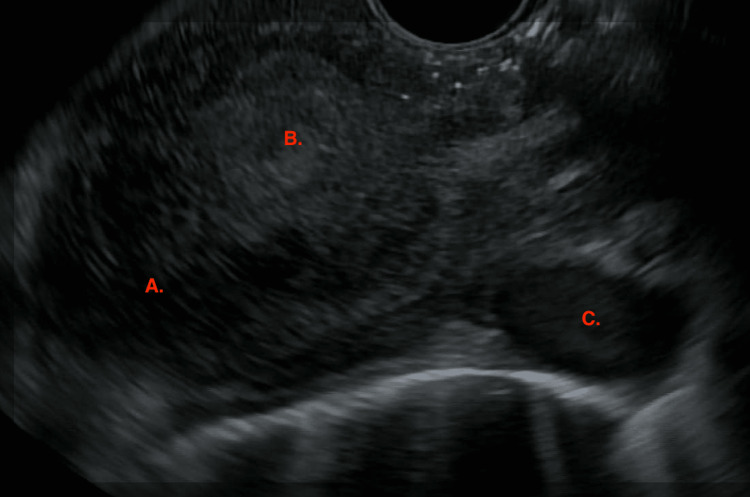
Endoscopic Ultrasound: partially circumferential mass involving the mucosa. A: Esophageal lumen B: Mass C: Malignant lymph node

**Figure 4:**
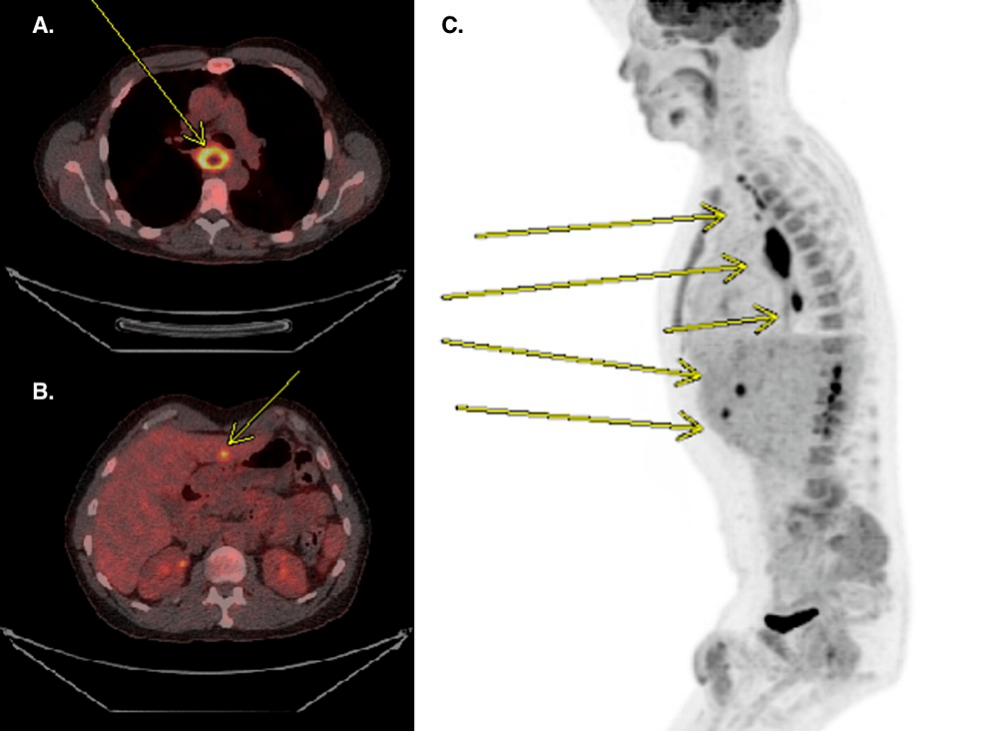
PET-CT A: circumferential mid-esophageal mass approximating 3 cm in diameter and 6 cm in length, SUV 8.7 units B: Liver metastasis C: Abnormal metabolically active lesions consistent with disease in the esophagus, subcarinal and paraesophageal lymph nodes

Mass biopsy demonstrated high-grade neuroendocrine carcinoma, with 90% of tumor cells being positive on the Ki-67 proliferation index. Immunohistochemical staining confirmed neuroendocrine carcinomatous differentiation with positive synaptophysin (Figure 2), CD56, and CAM5.2. Endoscopic ultrasonography showed the mass extending between 25 and 31 cm from the incisors with a muscularis propria invasion (Figure 3). PET-scan (Figure 4) demonstrated increased uptake in the mid-esophagus and regional lymph nodes with 2 foci of hepatic metastases, staging it as cT2N2M1.

He received palliative radiotherapy to the esophageal mass and was started on chemotherapy with carboplatin, etoposide, and atezolizumab immunotherapy. After completing four treatment cycles, he had a repeat CT scan which showed improved but persistent esophageal wall thickening with a reduction in the size of the mediastinal lymph nodes and liver metastasis.

## Discussion

Pure neuroendocrine carcinomas of the esophagus are exceedingly rare. A study of 8305 neuroendocrine tumors in different anatomical locations published in 1997 had only 0.4% NET (neuroendocrine tumors) from the esophagus [3]. A Korean study of 4951 gastroenteropancreatic NET had 1.4% NET arising from the esophagus. Although the overall incidence of gastroenteropancreatic NEC is rising, E-NEC is still rare [4-5].

There are no studies on risk factors for E-NEC, but previous studies consistently showed E-NEC to be diagnosed predominantly in male patients aged 40-60 and those with a history of smoking or chewing tobacco [3,5-8]. In our case, a high-grade pure neuroendocrine tumor without evidence of adenocarcinoma was found to be arising from Barrett's esophagus mucosa. Very few reports exist on pure esophageal neuroendocrine tumors arising from Barrett's mucosa [2,9].

More than half of E-NECs are metastasized by diagnosis [5,7-8]. E-NEC is staged using the AJCC TNM staging and grading systems with endoscopic ultrasound, Contrast Enhanced Computed Tomography (CECT), and Positron emission tomography-computed tomography (PET-CT) [10]. Definitive diagnosis of E-NEC can only be done using immunohistochemistry [11].

Treatment approaches include surgical resection combined with chemotherapy and radiotherapy. Surgery may benefit patients with limited-stage E-NEC only [6]. Meta-analysis and multicenter studies have shown that chemotherapy is the cornerstone of treatment in all stages of E-NEC. Concurrent chemo-radiotherapy has shown to be more effective than chemotherapy or surgery alone [6,12-13].

Previous studies on prognosis for esophageal neuroendocrine tumors showed that tumor size >2cm and metastatic disease are associated with poor prognosis and significant survival differences [6,14].

## Conclusions

Pure neuroendocrine carcinomas of the esophagus are extremely rare, comprising only a small percentage of neuroendocrine tumors in different anatomical locations. The pathogenesis remains unclear, and studies have consistently shown a higher prevalence in male patients aged 40-60 with a history of smoking or tobacco use. Barrett's mucosa possibly sets the stage for the development of E-NEC, even in the absence of adenocarcinoma. Concurrent chemo-radiotherapy is an effective treatment to reduce tumor burden, but still, these carcinomas involve poor prognosis, especially metastatic disease. Further research is required to establish clear risk factors, pathogenesis, and treatment strategies.
